# The Roles of Mutation and Selection Acting on Mitochondrial Genomes Inferred from Intraspecific Variation in Seed Plants

**DOI:** 10.3390/genes13061036

**Published:** 2022-06-09

**Authors:** Shenglong Kan, Xuezhu Liao, Zhiqiang Wu

**Affiliations:** 1 Shenzhen Branch, Guangdong Laboratory for Lingnan Modern Agriculture, Genome Analysis Laboratory of the Ministry of Agriculture, Agricultural Genomics Institute at Shenzhen, Shenzhen 518120, China; kanshenglong@caas.cn (S.K.); liaoxuezhu@caas.cn (X.L.); 2 Kunpeng Institute of Modern Agriculture at Foshan, Foshan 528200, China

**Keywords:** single-nucleotide polymorphisms, mutation rate, copy number variation, organelle, seed plants

## Abstract

There is a paradox in the plant mitochondrial genome, that is, the genic region evolves slowly while the intergenic region evolves rapidly. Thus, the intergenic regions of the plant mitochondrial genome are difficult to align across different species, even in closely related species. Here, to character the mechanism of this paradox, we identified interspecific variations in the *Ginkgo biloba*, *Oryza sativa*, and *Arabidopsis thaliana* mitochondrial and plastid genome at a genome-wide level. The substitution rate of synonymous sites in genic regions was similar to the substitution rate of intergenic regions, while the substitution rate of nonsynonymous sites in genic regions was lower than that in intergenic regions, suggesting the mutation inputs were the same among different categories within the organelle genome, but the selection pressure varied. The substitution rate of single-copy regions was higher than that of IR (inverted repeats) in the plastid genome at an intraspecific level. The substitution rate of single-copy regions was higher than that of repeats in the *G. biloba* and *A. thaliana* mitochondrial genomes, but lower in that of *O. sativa*. This difference may be related to the length and distribution of repeats. Copy number variations that existed in the *G. biloba* and *O. sativa* mitochondrial genomes were confirmed. This study reveals the intraspecific variation pattern of organelle genomes at a genome-wide level, and that copy number variations were common in plant mitochondrial genomes.

## 1. Introduction

The typical plant cell contains three relatively independent pieces of genetic information, including the nuclear genome, mitochondrial genome (mitogenome), and plastid genome. Compared with the intricated nuclear genome, the organelle genome is smaller and more suitable for investigating mutation mechanisms. Although the plastid and mitogenomes of seed plants are uniparentally inherited, in the high-energy environment, their synonymous substitution rates are far below than that of the nuclear genome and they differ significantly among different lineages [1]. The synonymous substitution rate of the plastid genome was three times higher than that of the mitogenome in angiosperms, while the synonymous substitution rate of the plastid genome was two times higher than that of the mitogenome in gymnosperms [2]. Exceptionally, the synonymous substitution rate of the mitogenome in some lineages (e.g., *Silene*, *Plantago*, *Pelargonium*, and *Ajuga*) have experienced highly accelerated substitution rates, even 100-fold higher than that in closely related species, as well as the synonymous substitution rate of the plastid genome in those lineages being coincidentally accelerated [3,4,5,6,7,8,9,10,11]. However, the potential mechanism for the mutation of the organelle genome is still poorly understood.

In contrast to the slow synonymous substitution rate in the coding region, the plant mitogenome is notorious for its rapid variation in the noncoding region [12,13]. Plant mitogenomes range from 66 Kb to 12.4 Mb in size, and contain a large number of repeat sequences and unknown derived sequences [14,15]. Due to recombination and rearrangement, the mitogenome exhibits linear, branched, and circular structures or subgenomes in vivo [16,17]. Several different hypotheses have been proposed for this phenomenon of the plant mitogenome. Christensen [18] compared the complete mitogenome of two ecotypes of *Arabidopsis thaliana*, and proposed that the repair mechanism accounts for the differences between the coding region and noncoding regions. However, Sloan, et al. [19] revealed that the old version of the *A. thaliana* mitogenome contains many sequencing errors that may lead to inaccurate analysis. Subsequently, by comparing the transcribed region and non-transcribed region of a small part of the mitogenome among some legumes, Christensen [20] examined that selection is the main factor responsible for the differences between the coding region and intergenic region. To reconcile the paradox of the plant mitogenome at a complete genome-wide level, Wu, et al. [21] analyzed intraspecific variation of *A. thaliana* from the *Arabidopsis* 1001 Genome Project, and supported that selection is the main factor. However, those conclusions are mainly based on the investigation of *A. thaliana* or a small part of the mitogenome, and may not be general for many other species. Additionally, although previous studies have suggested that the size and structure of the plastid genome is less variable than that of the mitochondria, the size and structure of the plastid genome also varies among different taxa, and IR (inverted repeats) region expansion, contraction, or loss frequently occurs, even in close relatives [22,23,24]. Therefore, more distant taxa need to be studied, and the variation mechanism of the plastid genome also needs to be paid attention to.

The rate of neutral substitutions is expected to equal the mutation rate, which is the fundamental premise of the neutral evolution theory [25]. Although synonymous substitutions may be selected by mRNA stability, translation efficiency, etc., and intergenic regions always contain mutational hotspots, they are always considered to be nearly neutral [25]. Thus, the substitution rates of synonymous sites and intergenic regions reflected the mutation input, whereas the nonsynonymous substitution rate always mirrored the degree of selective pressure. There were many methods for estimating the organelle substitution rate including phylogenetic methods, mutation accumulation lines, and population genetics [26,27]. Previous studies have chosen the phylogenetic method to estimate the substitution rate according to the DNA sequence among extant species, but substitution saturation may potentially affect the estimated substitution rate [4,6,8,28]. Due to the large variation in intergenic regions of plant organelle genomes, the intergenic regions of plant mitogenomes are especially characterized by a large amount of unknown origin sequences and repeats, which makes it difficult to compare it to the intergenic regions among species, even in closely related species [29]. Therefore, the phylogenetic methods only estimate the substitution rate of a small part of the plant mitogenome. By contrast, whole genome resequencing of mutation accumulation lines provides a chance to identify genome-wide variations in a defined number of generations in the laboratory. This method has been applied to estimate the substitution rate of yeast, *Drosophila*, and *Caenorhabditis* [30,31]. When Wu, et al. [21] applied this method to estimating the variations of the mitogenome in mutation accumulation lines of *A. thaliana* after propagating 10 generations, there were no true variations under manual inspection. Compared with yeast and *Drosophila*, plants have both long generation times and a low substitution rate of the organelle genome. Thus, the mutation accumulation lines method does not seem to be applicable in plant organelles. Barnard-Kubow, et al. [27] successfully employed the population genetics method to assess the intraspecific polymorphism in the plastid genome of *Campanulastrum americanum*. In addition, Wu, et al. [21] estimated the variation of the mitogenome in *A. thaliana* at an intraspecific level. Therefore, population genetics may be the most compatible method to assess an intraspecific polymorphism of the plant organelle genome.

In this study, we analyzed the intraspecific variation of the organelle genomes of *Ginkgo biloba*, *Oryza sativa*, and *A. thaliana*, representing gymnosperms, monocots, and dicots, respectively. Furthermore, the driving factors of the differences in substitution rates between genic and intergenic regions were studied. Moreover, copy number variations within the mitogenomes were investigated.

## 2. Materials and Methods

### 2.1. Data Resource

To investigate the intraspecific variation of organelle genomes in different clades of seed plants, we selected *G. biloba*, *O. sativa*, and *A. thaliana*, of which population genomic data have been released, as representatives of gymnosperms, monocots, and dicots, respectively. The total genomic DNA sequencing data of 312 *G. biloba* individuals (PRJNA478810), 40 *O. sativa* individuals (PRJEB19404), and 1135 *A. thaliana* (PRJNA273563) individuals were downloaded from the NCBI (National Center for Biotechnology Information, https://www.ncbi.nlm.nih.gov/, accessed on 1 November 2020) (Table S1) [32,33,34]. The sequence and its annotation files of mitochondrial and plastid genomes of *G. biloba* (NC_027976.1 and NC_016986.1), *O. sativa* (NC_011033.1 and NC_001320.1), and *A. thaliana* (NC_037304.1 and NC_000932.1) were also downloaded from the NCBI.

### 2.2. Identification of Intraspecific Variation in Organelle Genomes

The pipeline of the identification of intraspecific variation in organelle genomes followed Wu, et al. [21]. In order to reduce the impact of sequencing errors on the results, homologous sequences between the plastid and mitogenome, and homologous sequences between organelles and the nuclear genome, were underwent a series of strict quality control measures. The specific analysis process is as follows: First, Fastp v0.20.0 was used to perform quality control on the raw data of each accession, including removal of adapter sequences and low-quality bases [35]. Second, the MEM algorithm of BWA v0.7.17-r1188 was used to map the clean reads of each accession back to the mitochondrial and plastid reference genomes, and the primary SAM files were obtained [36]. Then, the SortSam tool included in GATK v4.0.12 was used to sort the primary SAM files by coordinate and convert them to BAM files. This was followed by indexing under the SAMtools v1.9 index command [37,38]. Third, the MarkDuplicates tool was used to locate and tag duplicate reads originating from a single DNA fragment, HaplotypeCaller, GenotypeGVCFs and SelectVariants tools were used to call SNPs and indels simultaneously from each accession. Subsequently, the VariantFiltration tool was used to hard-filter variant calls based on our strict criteria (QUAL < 60, QD < 20.0, FS > 10.0, MQ < 30.0) [37]. The filtered vcf file of each accession was merged into a total file using bcftools v1.9, and then a hard filter was performed again using the VariantFiltration tool to obtain the final results [37,39]. Finally, the ‘-TsTv-summary’ command of vcftools v0.1.16 was used to calculate a simple summary of all transition and transversion sites, and the ‘--freq2’ command was used to calculate the allele frequencies of each SNP and indel site [40].

### 2.3. Annotation of Intraspecific Variation in Organelle Genomes

Based on the annotation files of the mitochondrial and plastid reference genomes, we extracted the site information of functional classifications, such as coding and noncoding regions of protein-coding genes, rRNA genes, tRNA genes, pseudogenes, repeats, etc. For the coding region of protein-coding genes, PAML v4.9 was used to classify them into non-synonymous substitution sites and synonymous substitution sites, according to the degeneracy of the codons [41].

According to the above functional partitions, we annotated the intraspecific variation sites (SNPs and indels) of the mitochondrial and plastid genomes obtained in Section 2.2.

### 2.4. Estimation of Copy Number Variations within the Mitogenome

The copy number variation within the mitogenomes of *G. biloba* and *O. sativa* were estimated by calculating the mean coverage depth of non-overlapping windows of 1000 bp [21]. The analysis process is as follows: The ‘depth’ command of SAMtools v1.9 was used to process the sorted and indexed bam files in Section 2.2, obtain the sequencing depth of each site, and calculate the mean sequencing depth of each 1000 bp window [38]. Then, the maps of the mean sequencing depth of *G. biloba* and *O. sativa* were plotted by using the R v3.6.3 software.

## 3. Results

### 3.1. Intraspecific Variations in Mitochondrial Sequence

The intraspecific variations in mitochondrial sequence among 312 accessions of *G. biloba*, 40 accessions of *O. sativa*, and 1135 accessions of *A. thaliana* were identified at the genome-wide level. Although the number of accessions of the three species differed greatly, especially in *O. sativa* where there were only 40 accessions, but in order to cover the genetic diversity, samples from different populations were selected for analysis. Even though the size and representativeness affect the number of variations, the pattern among the different functional classifications within the organelle genome does not change.

**Figure 1:**
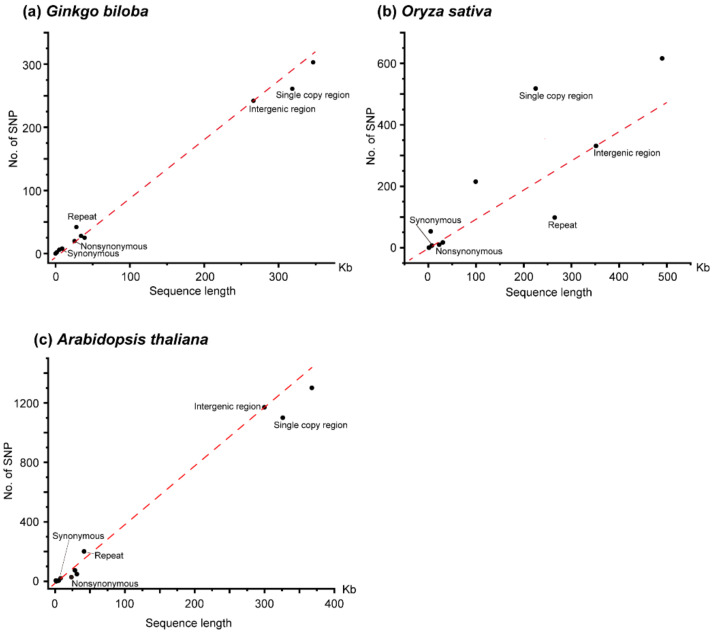
Scatter plot of length and number of SNPs for different categories within mitogenome. (**a**) *Ginkgo biloba*, (**b**) *Oryza sativa*, (**c**) *Arabidopsis thaliana*. The red dotted line indicates the relationship between length and the number of SNPs in intergenic region. Nonsynonymous and synonymous indicates nonsynonymous sites and synonymous sites of the coding regions of protein-coding genes, respectively.

**Figure 2:**
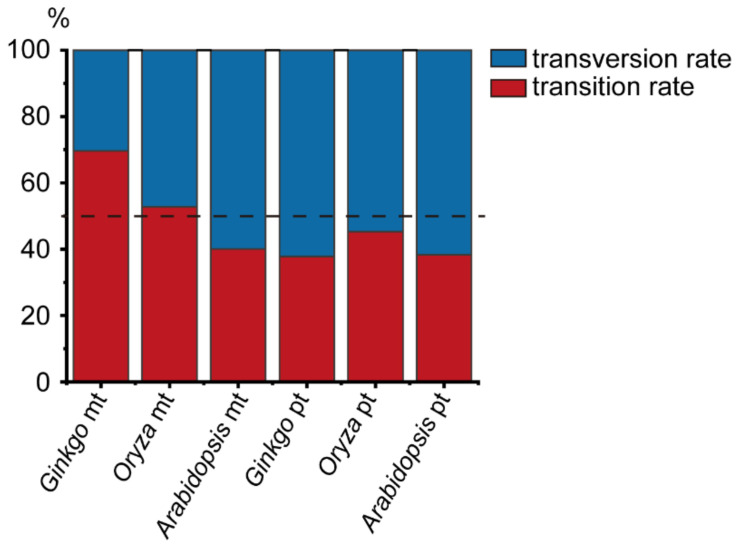
Percentage of transition and transversion rates within the genome of organelles in three species. The mt indicates mitogenome; pt indicates plastid genome.

A total of 303 high confidence SNPs (single nucleotide polymorphisms) and 139 indels (insertions and deletions) were identified across the mitogenome of *G. biloba* (Table 1). According to the function of the encoding products, the mitogenome of *G. biloba* was divided into coding regions of protein-coding genes, rRNA genes, tRNA genes, introns, and intergenic regions, and the lengths were 34,407 bp, 5006 bp, 1746 bp, 39,183 bp, and 266,202 bp, respectively. The coding regions of the protein-coding genes were also divided into nonsynonymous sites (25,519 bp) and synonymous sites (8888 bp). The number of SNPs in the above classifications were 28, 6, 2, 25, and 242. There were 20 and eight SNPs in nonsynonymous sites and synonymous sites of the coding regions of the protein-coding genes, respectively. Due to the different length of each classification, the SNP abundance of each functional region was calculated, from high to low, as follows: rRNA genes (0.0012 SNPs per site), tRNA genes (0.0011), intergenic and synonymous sites (0.0009), nonsynonymous sites (0.0008), and introns (0.0006). The SNP abundance of synonymous substitution sites was consistent with intergenic regions and may represent neutral evolution (Table 1, Figure 1a). In addition, these results suggested that rRNA and tRNA genes were under slightly positive selection, while nonsynonymous sites and introns were under slightly purified selection, or nearly neutral evolution as well. A total of 293 SNPs were in the *G. biloba* mitogenome after excluding the SNPs with more than two alleles, of which 204 SNPs were transitions and 89 SNPs were transversions, and the rate between transition and transversion was 2.29 (Table S2, Figure 2). Compared with the SNP content within the mitogenome, indels were only distributed in rRNA genes, introns, and intergenic regions, and their abundance was 0.0002 indels per site (one indel), 0.0003 (13 indels), and 0.0005 (125 indels), respectively. Additionally, as land plant mitogenomes usually contains a large number of repeats, we also calculated SNP and indel abundance of the single-copy sequence (318,455 bp) and repeats (28,089 bp), where SNP abundance was 0.0008 SNPs per site and 0.0015 SNPs per site, respectively, and indel abundance was 0.0004 indels per site, for both (Table 1). Compared with the SNP abundance of the intergenic region, single-copy sequence was more likely in neutral selection, while repeat sequences were under positive selection.

Compared to *G. biloba*, the introns and intergenic regions of the *O. sativa* mitogenome have expanded significantly, and two genes (*rps11* and *rps14*) became pseudogenes [42]. The length of nonsynonymous sites in the coding region of protein-coding genes, synonymous sites in the coding region of protein-coding genes, rRNA genes, tRNA genes, pseudogenes, introns, and intergenic regions in the *O. sativa* mitogenome were 23,009 bp, 7738 bp, 5324 bp, 1471 bp, 1561 bp, 99,594 bp, and 351,823 bp, and the number of SNPs in each classification was 10, 7, 53, 0, 0, 215, and 331, respectively (Table 1). The SNP abundance of each category in the *O. sativa* mitogenome was rRNA genes 0.0100 SNPs per site, introns 0.0022, intergenic regions and synonymous sites 0.0009, nonsynonymous sites 0.0004, and there were no SNPs in tRNA genes and pseudogenes. These results indicated that each category in the *O. sativa* mitogenome was under a different selection pressure; for example, the synonymous sites were under neutral selection, while the nonsynonymous sites were under purified selection (Table 1, Figure 1b). Among the confident SNPs, there were 597 SNPs with two alleles, including 315 transitions and 282 transversions, and the rate between transition and transversion was 1.12 (Table S2, Figure 2). In contrast to single base variations, indels mainly occurred in rRNA genes, introns and intergenic regions, suggesting that the non-coding regions were subject to a lower selection pressure. In the *O. sativa* mitogenome, the amount of single-copy sequence (225,262 bp) and repeats (265,258 bp) accounts for nearly half. However, there were far more SNPs in single-copy region (518 SNPs, 0.0023 SNPs per sites) than in repeats (98 SNPs, 0.0004 SNPs per sites) (Table 1). This suggested that the evolution rate of repeats was significantly slower than that of the single-copy region.

We also re-analyzed the intraspecific variations of the *A. thaliana* mitogenome by our analysis pipeline. As we did not exclude accessions with the length of clean reads shorter than 50 bp, we obtained slightly more variations than that in Wu, et al. [21] Compared with the *G. biloba* and *O. sativa* mitogenomes, the mitogenome of *A. thaliana* was similar to *G. biloba* in size, the length of intron, and the content of repeat sequence. The mitogenome of *A. thaliana* also encodes pseudogenes (*matR*). We also divided the mitogenome of *A. thaliana* into seven categories, and the length of nonsynonymous sites, synonymous sites, rRNA genes, tRNA genes, pseudogenes, introns, and intergenic regions were 23,381 bp, 7891 bp, 5222 bp, 1689 bp, 1256 bp, 28,422 bp, and 299,947 bp, respectively. We obtained 1,301 high confident SNPs from the mitogenome of *A. thaliana*, and there were 28, 20, 4, 0, 5, 74, and 1,170 SNPs in the above categories, respectively (Table 1). According to the SNP abundance, the order from the largest to smallest was pseudogenes (0.0040 SNPs per site), intergenic regions (0.0039), introns (0.0026), synonymous sites (0.0025), nonsynonymous sites (0.0012), rRNA genes (0.0008), and tRNA genes (0.0000). This suggested that the pseudogene was from nearly neutral evolution, while nonsynonymous sites, rRNA genes, and tRNA genes were under strong positive selection, and intron and synonymous sites were under slightly positive selection (Table 1, Figure 2). There were 519 transitions and 775 transversions in *A. thaliana*, and the rate between transition and transversion was much lower than that in *G. biloba* and *O. sativa* (Table S2, Figure 2). Compared with SNPs, there were only 220 indels, and they were mainly distributed in introns and intergenic regions, suggesting that indels were under a stronger purifying selection in the coding regions (Table 1). The total length of repeat sequences in the *A. thaliana* mitogenome was only 41,605 bp, accounting for 11.31%, while the total length of single-copy sequences was 326,203 bp, accounting for (88.69%). A total of 201 SNPs were identified from repeats with an abundance of 0.0048 SNPs per site, while 1,100 SNPs were obtained from the single-copy sequence with an abundance of 0.0034. The SNP abundance of single-copy sequence was similar to the intergenic regions, but the SNP abundance of repeats was larger than that of the intergenic region.

### 3.2. Intraspecific Variation in the Plastid Sequence

To compare the mutation pattern between the mitochondrial and plastid genomes, the intraspecific genetic diversity of the *G. biloba*, *O. sativa*, and *A. thaliana* plastid genomes were also analyzed at a genome-wide level. Since the typical structure of seed plants plastid genomes were tetrad-structures, including LSC (large single-copy region), SSC (small single-copy region), and IR (inverted repeat regions), and previous studies have shown that the evolution rate of IR regions is slower than that of single-copy regions (LSC and SSC) at an interspecific level [43], not only the nucleotide polymorphisms of the entire plastid genome was analyzed, but those of LSC, SSC, and IR were also analyzed, respectively. Similar to the analysis of the mitogenome, the entire plastid genome, LSC, SSC, and IR were also divided into seven functional categories: nonsynonymous sites in the coding region of protein-coding genes, synonymous sites in the coding region of protein-coding genes, rRNA genes, tRNA genes, pseudogenes, introns, and intergenic regions.

In the *G. biloba* plastid genome, the length of nonsynonymous sites, synonymous sites, rRNA genes, tRNA genes, introns, and intergenic regions were 50,043 bp, 16,305 bp, 8925 bp, 10,778 bp, 6523 bp, and 64,414 bp, respectively. Some 31, 11, 4, 5, 3, and 98 high confident SNPs were identified in the above categories, respectively (Table 2). According to the SNP abundance of each category from high to low, the order was as follows: intergenic regions (0.0015 SNPs per site), synonymous sites (0.0007), nonsynonymous sites (0.0006), tRNA genes and introns (0.0005), and rRNA genes (0.0004). The SNP abundance of synonymous sites was similar to nonsynonymous sites, and both were much lower than that of the intergenic regions, suggesting that both synonymous sites and nonsynonymous sites were under a strong purifying selection. The SNP abundance in the intergenic regions of IR was 0.0005, which was much lower than that of LSC (0.0023) and SSC (0.0022). In the LSC region, the SNP abundance of nonsynonymous sites (0.0005) was equal to that of synonymous sites, and both were lower than that of the intergenic regions, suggesting the nonsynonymous and synonymous sites were both under strong selection. However, in the SSC region, the SNP abundance of synonymous sites (0.0020) was similar to that of the intergenic regions (0.0022), but larger than that of nonsynonymous sites (0.0013). This indicated that the nonsynonymous sites and synonymous sites in the protein-coding region were under a different selective pressure. As there were only 723 synonymous sites in the IR region, there was no polymorphism site. Interestingly, the SNP abundance of nonsynonymous sites of the inverted repeats was higher than that of the intergenic regions, so nonsynonymous sites may experience positive selection. Furthermore, there were 56 transitions and 92 transversions, with a rate between transition and transversion of 0.61 (Table S2, Figure 2). Additionally, there were 5, 3, and 49 indels in the protein-coding regions, tRNA genes and intergenic regions, respectively (Table 2). These indicated that the protein-coding regions were under a stronger selection pressure than that of intergenic regions.

Compared with the *G. biloba* plastid genome, the size of the *O. sativa* plastid genome was much smaller, and the LSC and SSC regions were contracted, while the IR regions were expanded. Overall, the length of the nonsynonymous sites, synonymous sites, rRNA genes, tRNA genes, pseudogenes, introns, and intergenic regions were 44,295 bp, 14,524 bp, 9182 bp, 9346 bp, 883 bp, 10,615 bp, and 45,681 bp, respectively (Table 2). We identified 36, 24, 0, 1, 1, 5, and 64 high confident SNPs in the above categories. The SNP abundance of synonymous sites was 0.0017 SNPs per site, which was the highest across the plastid genome of *O. sativa*, slightly higher than that of intergenic region (0.0014), which means that it may experience nearly neural or mild selection. In contrast to the SNP abundance of introns (0.0005), that of pseudogenes (0.0011) were more closed to that of intergenic regions, suggesting introns may be under a stronger selection than pseudogenes. The SNP abundance of nonsynonymous sites (0.0008) was lower than that of synonymous sites and intergenic regions, indicating that the nonsynonymous sites were under a strong purifying selection. Besides, there were little polymorphism sites in the rRNA and tRNA genes. Surprisingly, there were no single base substitution sites in the IR region, either in the coding regions (i.e., nonsynonymous sites, synonymous sites) or intergenic regions. This suggested that the IR region may be in a very low evolution rate. Whether in LSC or SSC, the SNP abundance of the intergenic regions was greater than that of synonymous sites and much greater than that of nonsynonymous sites, indicating that synonymous sites and nonsynonymous sites were under a different purifying selective pressure. In the *O. sativa* plastid genome, there were 58 transition sites and 70 transversion sites, and the rate between transition and transversion was 0.83, which is higher than that of the *G. biloba* plastid genome (Table S2, Figure 2). In total, 110 indels were obtained, consistent with the pattern of *G. biloba*, with indels predominantly occurring in introns and intergenic regions (Table 2).

The size of the *A. thaliana* plastid genome is larger than that of *O. sativa,* and close to that of *G. biloba*. Compared with the *G. biloba* plastid genome, the IR regions were expanded and the single-copy regions (LSC and SSC) were contracted in the *A. thaliana* plastid genome. The complete plastid genome of *A. thaliana* was divided into nonsynonymous sites, synonymous sites, rRNA genes, tRNA genes, introns and intergenic regions, and the length of each category was 60,108 bp, 19,119 bp, 8929 bp, 10,233 bp, 12,711 bp, and 43,378 bp, respectively (Table 2). A total of 2330 high confident SNPs were identified, of which 376, 463, 1, 70, 217, and 1203 SNPs were in the above categories, respectively. The SNP abundance of each functional category from high to low was intergenic regions (0.0277 SNPs per site), synonymous sites (0.0242), introns (0.0171), tRNA genes (0.0068), nonsynonymous sites (0.0063), and rRNA genes (0.0001). As intergenic regions were in neutral evolution, the nonsynonymous sites may be under a strong purifying selection. The SNP abundance in the intergenic regions of LSC, SSC, and IR were 0.0581, 0.0471, and 0.0001. In LSC and SSC, the SNP abundance of synonymous sites was lower than that of the intergenic regions, and much higher than those of nonsynonymous sites. These indicated that the synonymous sites were under a mild purifying selection, while the nonsynonymous sites were under a much intensive selection. In the plastid genome of *A. thaliana*, there were 519 transition sites and 775 transversion sites, and the rate between transition and transversion is 0.62, which is lower than that of the *O. sativa* plastid genome (Table S2, Figure 2). In contrast to a single base nucleotide substitution, we obtained 695 indels in the *A. thaliana* plastid genome, and found that, similar to *G. biloba* and *O. sativa*, indels were mainly distributed in introns and the intergenic regions (Table 2).

### 3.3. Copy Number Variation within the Mitogenome

**Figure 3:**
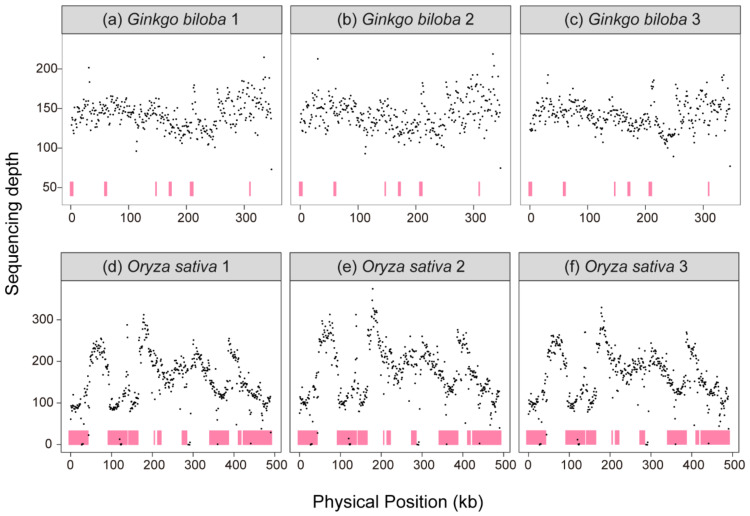
Sequencing depth of mitogenome. (**a**) *Ginkgo biloba* individual 1, (**b**) *G. biloba* individual 2, (**c**) *G. biloba* individual 3, (**d**) *Oryza sativa* individual 1, (**e**) *O. sativa* individual 2, (**f**) *O. sativa* individual 3. Pink color indicates the repeat sequences in mitogenomes.

To verify the copy number variations within the mitogenome, three samples of *G. biloba* and *O. sativa* were randomly selected to calculate their sequencing depth, respectively. Compared with the copy number variations, based on three purified mtDNA (mitochondrial DNA) samples of *A. thaliana* and validated by ddPCR (Droplet Digital PCR) [21], the sequencing depth variations within the *G. biloba* and *O. sativa* mitogenomes were also observed, suggesting copy number variations still existed in the *G. biloba* (Figure 3a–c) and *O. sativa* mitogenomes (Figure 3d–f).

## 4. Discussion

### 4.1. Evolution Rates in Genic and Intergenic Regions of Plant Organelle Genomes

We quantitatively compared the difference of intraspecific variations among different categories within organelle genomes by sampling multiple individuals of three species (gymnosperms, *G. biloba*; monocots, *O. sativa*; and dicots, *A. thaliana*) from different plant lineages. Because the effective population size, generation time, and life history of the three species are different, we cannot directly compare the number of variations among them. Therefore, the differences in the evolutionary rates between genic and intergenic regions of organelle genomes in *G. biloba*, *O. sativa*, and *A. thaliana* were compared at the intraspecific level. Except for the plastid genome of *G. biloba*, the mitogenomes of *G. biloba*, *O. sativa,* and *A. thaliana*, as well as the plastid genomes of *O. sativa* and *A. thaliana*, have the same or a similar substitution level between synonymous sites and intergenic regions, suggesting that the mutation rate of different functional categories within the organelle genomes were the same [18,20,21,27,44]. Meanwhile, the substitution rate of nonsynonymous sites was lower than that of intergenic regions in both mitochondrial and plastid genomes. These results are consistent with the traditional perspective that the mutation rates were the same both in genic and intergenic regions, but the selective pressure varied in genic and intergenic regions [20].

Although the substitution level of synonymous sites was close to that of the intergenic regions in *A. thaliana* mitochondrial and plastid genomes, it was still slightly lower than that of the intergenic regions. Moreover, the substitution level of synonymous sites was significantly lower than that of the intergenic regions in the *G. biloba* plastid genome, but close to that of nonsynonymous sites. These results indicated that the synonymous sites were also under different degrees of purifying selection in different species. Two explanations have been proposed for this observed phenomenon. First, synonymous substitution does not change the amino acid sequence, but its change will affect GC content, mRNA structure and stability, translation efficiency, and protein folding and solubility [45,46,47,48,49]. Second, synonymous site substitution may be repaired, along with the repair of their adjacent nonsynonymous sites, by gene conversion [50,51,52]. Unexpectedly, the substitution rate of synonymous sites was slightly higher than that of the intergenic regions in the *O. sativa* plastid genome, and was 2.1 times higher than that of nonsynonymous sites, implying that the synonymous sites were under positive selection, and were not coupled to the evolution of genes. This phenomenon had also been reported in mammalian and bacterial nuclear genomes [45,53]. The synonymous sites of about 12% of genes were under positive selection in mammals. Although the substitutions of synonymous sites fixed under positive selection would affect mRNA stability, they may increase mRNA expression and translation to maintain physiological activity [45,53].

### 4.2. Evolution Rates in Repeats and Single-Copy Regions of Plant Organelle Genomes

The substitution rate of the intergenic regions of IR in the *G. biloba*, *O. sativa*, and *A. thaliana* plastid genomes were lower than that of the single-copy regions (LSC and SCC), which was consistent with a large number of previous studies in land plants [23,54,55,56]. The two IR regions in the plastid genome were identical in sequence, but in opposite directions, which provided a more homologous sequence than the single-copy regions for gene conversion to maintain their sequence identity, and they exhibited a lower mutation rate [23,57]. This explanation had been proven by direct experimental evidence [58]. However, there was more complexity when compared to the substitution rate of repeat sequences with that of single-copy sequences in the *G. biloba*, *O. sativa*, and *A. thaliana* mitogenomes. The substitution rate of repeats in *O. sativa* was lower than that of single-copy sequences, while the substitution rate of repeats in *G. biloba* and *A. thaliana* was higher than that of the single-copy sequence. Since the *O. sativa* mitogenome contains a large number of repeats, and the total length of repeats accounts for 54.1% [42], the mechanism that maintained the substitution rate of the repeats was lower than that of the single-copy regions, which may be consistent with the plastid genome. On the contrary, the repeats were less than 10% in both the *G. biloba* and *A. thaliana* mitogenomes and dispersed [50,59]. Additionally, the activity of repeats is related to their length [29,60], while the long repeats in the *G. biloba* and *A. thaliana* mitogenomes were rare, resulting in the frequency of homologous repair at a low level. Moreover, the repeat sequences were always in noncoding regions. Therefore, the pattern of variation between the repeats and single-copy regions in the *G. biloba* and *A. thaliana* mitogenomes are different from that of *O. sativa*.

The substitution rate of the intergenic regions and nonsynonymous sites in the *O. sativa* and *A. thaliana* plastid genomes were higher than that in the mitogenomes, which was consistent with the previous findings that the evolution rate of the plastid was faster than that of the mitochondria in land plants [1,2,61,62]. However, in *G. biloba*, the substitution rate of the intergenic regions of the plastid genome was higher than that of the mitogenome, while the nonsynonymous substitution rate of the plastid genome was slightly lower than that of the mitogenome; this suggested that the mutation rate of the plastid genome was higher than for the mitogenome, but the selection was stronger than that of the mitogenome. This result may be consistent with the previous study detailing that the *G. biloba* and cycad plastid genomes were in evolutionary stasis, but the mechanism remains unknown [55].

### 4.3. Copy Number Variations within Plant Mitogenomes and Implications for Genome Structure

We mapped the genomic sequencing short reads to the mitochondrial reference genomes of *G. biloba* and *O. sativa*, and the copy number variations across the mitogenome still found in them, which was consistent with the results of that in *A. thaliana* [21]. Illumina sequencing can rapidly quantify sequencing depth variations across the entire genome, but the sequencing depth is always affected by GC content and the complex structures of the sequence. In contrast, ddPCR is not affected by amplification efficiency and bias caused by GC content and the structure of sequences. Wu, et al. [21] mapped Illumina sequencing reads to the reference genome and validated by ddPCR. They found that the observed heterogeneity of the sequencing depth among three replicates was consistent, and six marks with high sequencing depth and six marks with low sequencing depth were validated by ddPCR, indicating that the copy number variation, based on Illumina sequencing data, was reliable. Frequency recombination or rearrangement of the mitogenome produced alternative conformations, and different stoichiometry among different conformations, manifesting as copy number variations in the master circle. For the most extreme example, the *Silene noctiflora* BRP mitogenome contains 63 chromosomes with a different sequencing depth [63]. The most common example is the *Mimulus guttatus* mitogenome, which contained many alternative subgenome-mediated recombination [64]. Additionally, there is increasing evidence that the structure of the mitogenome is much more complex than the simple master circle molecule, such as the multiple circular mitogenome in *Cucumis sativus* and *Lophophytum mirabile*, and the complex structure in *Picea sitchensis* and *Larix sibirica* [15,65,66,67]. Moreover, the physical structure of mtDNA molecules in *Lactuca sativa* were in branched, linear, and circular forms in fluorescence microscopy [17]. The structure of mtDNA molecules in *Vigna radiata* transformed from rosette-like to linear in vivo under different cold treatments [16]. All those evidence suggested that the copy number variation fluctuated within the mitogenome in different plants.

## 5. Conclusions

In this study, we analyzed the intraspecific variations of the organelle genomes of *G. biloba*, *O. sativa*, and *A. thaliana,* representing the gymnosperms, monocots, and dicots, respectively. We found that: (1) The mutation input in different functional categories (especially coding regions and intergenic regions) within organelle genomes were the same or similar, suggesting that selection contributed to the varied evolution rate among different functional categories. (2) Since the repeat sequences provided more material for the homology repair mechanism (gene conversion), the evolution rate of the IR region in plastid genomes was slower than that of the single-copy region. Because the *O. sativa* mitogenome also contains a large number of repeats, the evolution rate of repeats was lower than that of the single-copy regions. (3) The copy number variation within the mitogenome was common. This study sampled representative species from different clades of seed plants (i.e., gymnosperms, monocots, and dicots) and revealed the intraspecific variations pattern of the organelle genomes at a genome-wide level.

## Figures and Tables

**Table 1:** Intraspecific variations in mitogenomes s. SNPs: single nucleotide polymorphisms; MAF: minor allele frequency.

	Sequence Type	Sites	SNPs	SNPs per Site	SNP MAF	Indels	Indels per Site	Indel MAF
*Ginkgo biloba*	Protein Coding	34,407	28	0.0008	0.4606	0	0.0000	NA
	Nonsynonymous	25,519	20	0.0008	0.4850	0	0.0000	NA
	Synonymous	8888	8	0.0009	0.3996	0	0.0000	NA
	rRNA	5006	6	0.0012	0.1819	1	0.0002	0.1447
	tRNA	1746	2	0.0011	0.5000	0	0.0000	NA
	Pseudogene	0	0	NA	NA	0	0.0000	NA
	Intron	39,183	25	0.0006	0.3971	13	0.0003	0.2202
	Intergenic	266,202	242	0.0009	0.3917	125	0.0005	0.1930
	Single-copy region	318,455	261	0.0008	0.4369	127	0.0004	0.1984
	Repeat	28,089	42	0.0015	0.1351	12	0.0004	0.1611
	Total	346,544	303	0.0009	0.3951	139	0.0004	0.1952
*Oryza sativa*	Protein Coding	30,747	17	0.0006	0.0000	3	0.0001	0.0370
	Nonsynonymous	23,009	10	0.0004	0.0000	3	0.0001	0.0370
	Synonymous	7738	7	0.0009	0.0000	0	0.0000	NA
	rRNA	5324	53	0.0100	0.0189	26	0.0049	0.1026
	tRNA	1471	0	0.0000	NA	0	0.0000	NA
	Pseudogene	1561	0	0.0000	NA	0	0.0000	NA
	Intron	99,594	215	0.0022	0.1094	60	0.0006	0.0827
	Intergenic	351,823	331	0.0009	0.1652	92	0.0003	0.1712
	Single-copy region	225,262	518	0.0023	0.1462	145	0.0006	0.1296
	Repeat	265,258	98	0.0004	0.0355	36	0.0001	0.1306
	Total	490,520	616	0.0013	0.1286	181	0.0004	0.1298
*Arabidopsis thaliana*	Protein Coding	31,272	48	0.0015	0.0000	1	0.0000	0.0000
	Nonsynonymous	23,381	28	0.0012	0.0000	1	0.0000	0.0000
	Synonymous	7891	20	0.0025	0.0000	0	0.0000	NA
	rRNA	5222	4	0.0008	0.0000	0	0.0000	NA
	tRNA	1689	0	0.0000	NA	0	0.0000	NA
	Pseudogene	1256	5	0.0040	0.0000	0	0.0000	NA
	Intron	28,422	74	0.0026	0.0000	13	0.0005	0.0538
	Intergenic	299,947	1170	0.0039	0.0015	206	0.0007	0.0222
	Single-copy region	326,203	1100	0.0034	0.0016	173	0.0005	0.0202
	Repeat	41,605	201	0.0048	0.0000	47	0.0011	0.0378
	Total	367,808	1301	0.0035	0.0014	220	0.0006	0.0240

**Table 2:** Intraspecific variations in plastid genomes. SNPs: single nucleotide polymorphisms.

		*Ginkgo biloba*					*Oryza sativa*					*Arabidopsis thaliana*				
	Sequence Type	Sites	SNPs	SNPs per Site	Indels	Indels per Site	Sites	SNPs	SNPs per Site	Indels	Indels per Site	Sites	SNPs	SNPs per Site	Indels	Indels per Site
Total	Protein Coding	66,348	42	0.0006	5	0.0001	58,818	60	0.001	11	0.0002	79,227	839	0.0106	19	0.0002
	Nonsynonymous	50,043	31	0.0006	5	0.0001	44,295	36	0.0008	11	0.0002	60,108	376	0.0063	14	0.0002
	Synonymous	16,305	11	0.0007	0	0	14,524	24	0.0017	0	0	19,119	463	0.0242	5	0.0003
	rRNA	8925	4	0.0004	0	0	9182	0	0	0	0	8929	1	0.0001	2	0.0002
	tRNA	10,778	5	0.0005	3	0.0003	9346	1	0.0001	2	0.0002	10,233	70	0.0068	20	0.002
	Pseudogene	0	0	NA	0	NA	883	1	0.0011	0	0	0	0	NA	0	0
	Intron	6523	3	0.0005	4	0.0006	10,615	5	0.0005	28	0.0026	12,711	217	0.0171	77	0.0061
	Intergenic	64,414	98	0.0015	49	0.0008	45,681	64	0.0014	69	0.0015	43,378	1203	0.0277	577	0.0133
	Total	156,988	152	0.001	61	0.0004	134,525	131	0.001	110	0.0008	154,478	2330	0.0151	695	0.0045
LSC	Protein Coding	53,262	25	0.0005	4	0.0001	42,708	54	0.0013	5	0.0001	43,401	537	0.0124	14	0.0003
	Nonsynonymous	40,232	19	0.0005	4	0.0001	32,181	33	0.001	5	0.0002	32,720	190	0.0058	9	0.0003
	Synonymous	13,030	6	0.0005	0	0	10,527	21	0.002	0	0	10,681	347	0.0325	5	0.0005
	rRNA	0	0	NA	0	NA	0	0	0	0	0	0	0	0	0	0
	tRNA	6300	5	0.0008	3	0.0005	4559	1	0.0002	2	0.0004	6057	69	0.0114	19	0.0031
	Pseudogene	0	0	NA	0	NA	223	1	0.0045	0	0	0	0	0	0	0
	Intron	5774	3	0.0005	4	0.0007	5798	5	0.0009	27	0.0047	7823	196	0.0251	69	0.0088
	Intergenic	24,993	58	0.0023	43	0.0017	18,122	57	0.0031	67	0.0037	17,960	1043	0.0581	505	0.0281
	Total	99,254	91	0.0009	54	0.0005	80,592	118	0.0015	101	0.0013	84,170	1845	0.0219	607	0.0072
IR	Protein Coding	2892	2	0.0007	0	0	7542	0	0	0	0	22,542	9	0.0004	1	0
	Nonsynonymous	2169	2	0.0009	0	0	5672	0	0	0	0	17,249	9	0.0005	1	0.0001
	Synonymous	723	0	0	0	0	1870	0	0	0	0	5293	0	0	0	0
	rRNA	8925	4	0.0004	0	0	9182	0	0	0	0	8929	1	0.0001	2	0.0002
	tRNA	4324	0	0	0	0	4707	0	0	0	0	4096	1	0.0002	1	0.0002
	Pseudogene	0	0	NA	0	NA	660	0	0	0	0	0	0	0	0	0
	Intron	749	0	0	0	0	3830	0	0	0	0	3808	0	0	0	0
	Intergenic	27,502	14	0.0005	2	0.0001	24,859	0	0	1	0	22,082	3	0.0001	5	0.0002
	Total	35,467	20	0.0006	2	0.0001	41,598	0	0	1	0	52,528	14	0.0003	9	0.0002
SSC	Protein Coding	10,194	15	0.0015	1	0.0001	8568	6	0.0007	6	0.0007	13,284	293	0.0221	4	0.0003
	Nonsynonymous	7645	10	0.0013	1	0.0001	6448	3	0.0005	6	0.0009	10,139	177	0.0175	4	0.0004
	Synonymous	2549	5	0.002	0	0	2120	3	0.0014	0	0	3145	116	0.0369	0	0
	rRNA	0	0	NA	0	NA	0	0	0	0	0	0	0	0	0	0
	tRNA	154	0	0	0	0	80	0	0	0	0	80	0	0	0	0
	Pseudogene	0	0	NA	0	NA	0	0	0	0	0	0	0	0	0	0
	Intron	0	0	NA	0	NA	987	0	0	1	0.001	1080	21	0.0194	8	0.0074
	Intergenic	11,919	26	0.0022	4	0.0003	2700	7	0.0026	1	0.0004	3336	157	0.0471	67	0.0201
	Total	22,267	41	0.0018	5	0.0002	12,335	13	0.0011	8	0.0006	17,780	471	0.0265	79	0.0044

## Data Availability

Not applicable.

## Supplementary Materials

The following supporting information can be downloaded at: https://www.mdpi.com/article/10.3390/genes13061036/s1, Table S1. Accession number of samples used in this study; Table S2. Transition and transversion rate in the organelle genomes of each species.
