# Supplementary material for: The Roles of Mutation and Selection Acting on Mitochondrial Genomes Inferred from Intraspecific Variation in Seed Plants
Source: Genes (Basel). 2022 Jun 9;13(6):1036. doi: 10.3390/genes13061036 (PMC9222611; doi:10.3390/genes13061036)
Supplement: Supplementary file 1 [file genes-13-01036-s001.zip › genes-1725350-supplementary.pdf]

Table S1 accession number of samples used in this study.

Ginkgo biloba		***Arabisopsis thaliana***			***Oryza sativa***
SRR7648617	SRR1945435	SRR1945747	SRR1946059	SRR1946371	ERR2240124
SRR7648618	SRR1945436	SRR1945748	SRR1946060	SRR1946372	ERR2240127
SRR7648619	SRR1945437	SRR1945749	SRR1946061	SRR1946373	ERR2240128
SRR7648620	SRR1945438	SRR1945750	SRR1946062	SRR1946374	ERR2241054
SRR7648621	SRR1945439	SRR1945751	SRR1946063	SRR1946375	ERR2241055
SRR7648625	SRR1945440	SRR1945752	SRR1946064	SRR1946376	ERR2241056
SRR7648626	SRR1945441	SRR1945753	SRR1946065	SRR1946377	ERR2241057
SRR7648627	SRR1945442	SRR1945754	SRR1946066	SRR1946378	ERR2241058
SRR7648628	SRR1945443	SRR1945755	SRR1946067	SRR1946379	ERR2241059
SRR7648629	SRR1945444	SRR1945756	SRR1946068	SRR1946380	ERR2241060
SRR7648630	SRR1945445	SRR1945757	SRR1946069	SRR1946381	ERR2242620
SRR7648632	SRR1945446	SRR1945758	SRR1946070	SRR1946382	ERR2242621
SRR7648633	SRR1945447	SRR1945759	SRR1946071	SRR1946383	ERR2242622
SRR7648634	SRR1945448	SRR1945760	SRR1946072	SRR1946384	ERR2242624
SRR7648636	SRR1945449	SRR1945761	SRR1946073	SRR1946385	ERR2242626
SRR7648637	SRR1945450	SRR1945762	SRR1946074	SRR1946386	ERR2245516
SRR7648638	SRR1945451	SRR1945763	SRR1946075	SRR1946387	ERR2245517
SRR7648639	SRR1945452	SRR1945764	SRR1946076	SRR1946388	ERR2245518
SRR7648640	SRR1945453	SRR1945765	SRR1946077	SRR1946389	ERR2245519
SRR7648643	SRR1945454	SRR1945766	SRR1946078	SRR1946390	ERR2245520
SRR7648644	SRR1945455	SRR1945767	SRR1946079	SRR1946391	ERR2245523
SRR7648645	SRR1945456	SRR1945768	SRR1946080	SRR1946392	ERR2245524
SRR7648648	SRR1945457	SRR1945769	SRR1946081	SRR1946393	ERR2245525
SRR7648649	SRR1945458	SRR1945770	SRR1946082	SRR1946394	ERR2245527
SRR7648651	SRR1945459	SRR1945771	SRR1946083	SRR1946395	ERR2245528
SRR7648652	SRR1945460	SRR1945772	SRR1946084	SRR1946396	ERR2245529
SRR7648653	SRR1945461	SRR1945773	SRR1946085	SRR1946397	ERR2245530
SRR7648654	SRR1945462	SRR1945774	SRR1946086	SRR1946398	ERR2245531
SRR7648655	SRR1945463	SRR1945775	SRR1946087	SRR1946399	ERR2245533
SRR7648656	SRR1945464	SRR1945776	SRR1946088	SRR1946400	ERR2245534
SRR7648659	SRR1945465	SRR1945777	SRR1946089	SRR1946401	ERR2245535
SRR7648660	SRR1945466	SRR1945778	SRR1946090	SRR1946402	ERR2245536
SRR7648661	SRR1945467	SRR1945779	SRR1946091	SRR1946403	ERR2245537
SRR7648662	SRR1945468	SRR1945780	SRR1946092	SRR1946404	ERR2245538
SRR7648663	SRR1945469	SRR1945781	SRR1946093	SRR1946405	ERR2245539
SRR7648664	SRR1945470	SRR1945782	SRR1946094	SRR1946406	ERR2245543
SRR7648665	SRR1945471	SRR1945783	SRR1946095	SRR1946407	ERR2245544
SRR7648666	SRR1945472	SRR1945784	SRR1946096	SRR1946408	ERR2245545
SRR7648667	SRR1945473	SRR1945785	SRR1946097	SRR1946409	ERR2245546
SRR7648668	SRR1945474	SRR1945786	SRR1946098	SRR1946410	ERR2245547
SRR7648669	SRR1945475	SRR1945787	SRR1946099	SRR1946411	

SRR7648670	SRR1945476	SRR1945788	SRR1946100	SRR1946412
SRR7648672	SRR1945477	SRR1945789	SRR1946101	SRR1946413
SRR7648673	SRR1945478	SRR1945790	SRR1946102	SRR1946414
SRR7648674	SRR1945479	SRR1945791	SRR1946103	SRR1946415
SRR7648675	SRR1945480	SRR1945792	SRR1946104	SRR1946416
SRR7648677	SRR1945481	SRR1945793	SRR1946105	SRR1946417
SRR7648678	SRR1945482	SRR1945794	SRR1946106	SRR1946418
SRR7648679	SRR1945483	SRR1945795	SRR1946107	SRR1946419
SRR7648680	SRR1945484	SRR1945796	SRR1946108	SRR1946420
SRR7648681	SRR1945485	SRR1945797	SRR1946109	SRR1946421
SRR7648682	SRR1945486	SRR1945798	SRR1946110	SRR1946422
SRR7648683	SRR1945487	SRR1945799	SRR1946111	SRR1946423
SRR7648684	SRR1945488	SRR1945800	SRR1946112	SRR1946424
SRR7648686	SRR1945489	SRR1945801	SRR1946113	SRR1946425
SRR7648688	SRR1945490	SRR1945802	SRR1946114	SRR1946426
SRR7648689	SRR1945491	SRR1945803	SRR1946115	SRR1946427
SRR7648690	SRR1945492	SRR1945804	SRR1946116	SRR1946428
SRR7648691	SRR1945493	SRR1945805	SRR1946117	SRR1946429
SRR7648692	SRR1945494	SRR1945806	SRR1946118	SRR1946430
SRR7648693	SRR1945495	SRR1945807	SRR1946119	SRR1946431
SRR7648694	SRR1945496	SRR1945808	SRR1946120	SRR1946432
SRR7648696	SRR1945497	SRR1945809	SRR1946121	SRR1946433
SRR7648699	SRR1945498	SRR1945810	SRR1946122	SRR1946434
SRR7648700	SRR1945499	SRR1945811	SRR1946123	SRR1946435
SRR7648701	SRR1945500	SRR1945812	SRR1946124	SRR1946436
SRR7648702	SRR1945501	SRR1945813	SRR1946125	SRR1946437
SRR7648703	SRR1945502	SRR1945814	SRR1946126	SRR1946438
SRR7648704	SRR1945503	SRR1945815	SRR1946127	SRR1946439
SRR7648705	SRR1945504	SRR1945816	SRR1946128	SRR1946440
SRR7648706	SRR1945505	SRR1945817	SRR1946129	SRR1946441
SRR7648709	SRR1945506	SRR1945818	SRR1946130	SRR1946442
SRR7648710	SRR1945507	SRR1945819	SRR1946131	SRR1946443
SRR7648711	SRR1945508	SRR1945820	SRR1946132	SRR1946444
SRR7648717	SRR1945509	SRR1945821	SRR1946133	SRR1946445
SRR7648718	SRR1945510	SRR1945822	SRR1946134	SRR1946446
SRR7648721	SRR1945511	SRR1945823	SRR1946135	SRR1946447
SRR7648722	SRR1945512	SRR1945824	SRR1946136	SRR1946448
SRR7648724	SRR1945513	SRR1945825	SRR1946137	SRR1946449
SRR7648725	SRR1945514	SRR1945826	SRR1946138	SRR1946450
SRR7648726	SRR1945515	SRR1945827	SRR1946139	SRR1946451
SRR7648727	SRR1945516	SRR1945828	SRR1946140	SRR1946452
SRR7648728	SRR1945517	SRR1945829	SRR1946141	SRR1946453
SRR7648729	SRR1945518	SRR1945830	SRR1946142	SRR1946454
SRR7648730	SRR1945519	SRR1945831	SRR1946143	SRR1946455

SRR7648731	SRR1945520	SRR1945832	SRR1946144	SRR1946456
SRR7648733	SRR1945521	SRR1945833	SRR1946145	SRR1946457
SRR7648734	SRR1945522	SRR1945834	SRR1946146	SRR1946458
SRR7648740	SRR1945523	SRR1945835	SRR1946147	SRR1946459
SRR7648743	SRR1945524	SRR1945836	SRR1946148	SRR1946460
SRR7648744	SRR1945525	SRR1945837	SRR1946149	SRR1946461
SRR7648745	SRR1945526	SRR1945838	SRR1946150	SRR1946462
SRR7648746	SRR1945527	SRR1945839	SRR1946151	SRR1946463
SRR7648747	SRR1945528	SRR1945840	SRR1946152	SRR1946464
SRR7648748	SRR1945529	SRR1945841	SRR1946153	SRR1946465
SRR7648750	SRR1945530	SRR1945842	SRR1946154	SRR1946466
SRR7648751	SRR1945531	SRR1945843	SRR1946155	SRR1946467
SRR7648752	SRR1945532	SRR1945844	SRR1946156	SRR1946468
SRR7648753	SRR1945533	SRR1945845	SRR1946157	SRR1946469
SRR7648754	SRR1945534	SRR1945846	SRR1946158	SRR1946470
SRR7648756	SRR1945535	SRR1945847	SRR1946159	SRR1946471
SRR7648757	SRR1945536	SRR1945848	SRR1946160	SRR1946472
SRR7648758	SRR1945537	SRR1945849	SRR1946161	SRR1946473
SRR7648759	SRR1945538	SRR1945850	SRR1946162	SRR1946474
SRR7648761	SRR1945539	SRR1945851	SRR1946163	SRR1946475
SRR7648762	SRR1945540	SRR1945852	SRR1946164	SRR1946476
SRR7648763	SRR1945541	SRR1945853	SRR1946165	SRR1946477
SRR7648765	SRR1945542	SRR1945854	SRR1946166	SRR1946478
SRR7648766	SRR1945543	SRR1945855	SRR1946167	SRR1946479
SRR7648767	SRR1945544	SRR1945856	SRR1946168	SRR1946480
SRR7648768	SRR1945545	SRR1945857	SRR1946169	SRR1946481
SRR7648769	SRR1945546	SRR1945858	SRR1946170	SRR1946482
SRR7648771	SRR1945547	SRR1945859	SRR1946171	SRR1946483
SRR7648772	SRR1945548	SRR1945860	SRR1946172	SRR1946484
SRR7648773	SRR1945549	SRR1945861	SRR1946173	SRR1946485
SRR7648774	SRR1945550	SRR1945862	SRR1946174	SRR1946486
SRR7648775	SRR1945551	SRR1945863	SRR1946175	SRR1946487
SRR7648776	SRR1945552	SRR1945864	SRR1946176	SRR1946488
SRR7648777	SRR1945553	SRR1945865	SRR1946177	SRR1946489
SRR7648779	SRR1945554	SRR1945866	SRR1946178	SRR1946490
SRR7648781	SRR1945555	SRR1945867	SRR1946179	SRR1946491
SRR7648782	SRR1945556	SRR1945868	SRR1946180	SRR1946492
SRR7648783	SRR1945557	SRR1945869	SRR1946181	SRR1946493
SRR7648784	SRR1945558	SRR1945870	SRR1946182	SRR1946494
SRR7648785	SRR1945559	SRR1945871	SRR1946183	SRR1946495
SRR7648789	SRR1945560	SRR1945872	SRR1946184	SRR1946496
SRR7648790	SRR1945561	SRR1945873	SRR1946185	SRR1946497
SRR7648791	SRR1945562	SRR1945874	SRR1946186	SRR1946498
SRR7648792	SRR1945563	SRR1945875	SRR1946187	SRR1946499

SRR7648795	SRR1945564	SRR1945876	SRR1946188	SRR1946500
SRR7648796	SRR1945565	SRR1945877	SRR1946189	SRR1946501
SRR7648797	SRR1945566	SRR1945878	SRR1946190	SRR1946502
SRR7648798	SRR1945567	SRR1945879	SRR1946191	SRR1946503
SRR7648800	SRR1945568	SRR1945880	SRR1946192	SRR1946504
SRR7648801	SRR1945569	SRR1945881	SRR1946193	SRR1946505
SRR7648804	SRR1945570	SRR1945882	SRR1946194	SRR1946506
SRR7648806	SRR1945571	SRR1945883	SRR1946195	SRR1946507
SRR7648807	SRR1945572	SRR1945884	SRR1946196	SRR1946508
SRR7648809	SRR1945573	SRR1945885	SRR1946197	SRR1946509
SRR7648811	SRR1945574	SRR1945886	SRR1946198	SRR1946510
SRR7648812	SRR1945575	SRR1945887	SRR1946199	SRR1946511
SRR7648817	SRR1945576	SRR1945888	SRR1946200	SRR1946512
SRR7648818	SRR1945577	SRR1945889	SRR1946201	SRR1946513
SRR7648819	SRR1945578	SRR1945890	SRR1946202	SRR1946514
SRR7648821	SRR1945579	SRR1945891	SRR1946203	SRR1946515
SRR7648822	SRR1945580	SRR1945892	SRR1946204	SRR1946516
SRR7648824	SRR1945581	SRR1945893	SRR1946205	SRR1946517
SRR7648825	SRR1945582	SRR1945894	SRR1946206	SRR1946518
SRR7648826	SRR1945583	SRR1945895	SRR1946207	SRR1946519
SRR7648828	SRR1945584	SRR1945896	SRR1946208	SRR1946520
SRR7648829	SRR1945585	SRR1945897	SRR1946209	SRR1946521
SRR7648830	SRR1945586	SRR1945898	SRR1946210	SRR1946522
SRR7648831	SRR1945587	SRR1945899	SRR1946211	SRR1946523
SRR7648832	SRR1945588	SRR1945900	SRR1946212	SRR1946524
SRR7648833	SRR1945589	SRR1945901	SRR1946213	SRR1946525
SRR7648834	SRR1945590	SRR1945902	SRR1946214	SRR1946526
SRR7648835	SRR1945591	SRR1945903	SRR1946215	SRR1946527
SRR7648836	SRR1945592	SRR1945904	SRR1946216	SRR1946528
SRR7648837	SRR1945593	SRR1945905	SRR1946217	SRR1946529
SRR7648838	SRR1945594	SRR1945906	SRR1946218	SRR1946530
SRR7648839	SRR1945595	SRR1945907	SRR1946219	SRR1946531
SRR7648840	SRR1945596	SRR1945908	SRR1946220	SRR1946532
SRR7648843	SRR1945597	SRR1945909	SRR1946221	SRR1946533
SRR7648848	SRR1945598	SRR1945910	SRR1946222	SRR1946534
SRR7648852	SRR1945599	SRR1945911	SRR1946223	SRR1946535
SRR7648854	SRR1945600	SRR1945912	SRR1946224	SRR1946536
SRR7648855	SRR1945601	SRR1945913	SRR1946225	SRR1946537
SRR7648858	SRR1945602	SRR1945914	SRR1946226	SRR1946538
SRR7648860	SRR1945603	SRR1945915	SRR1946227	SRR1946539
SRR7648863	SRR1945604	SRR1945916	SRR1946228	SRR1946540
SRR7648866	SRR1945605	SRR1945917	SRR1946229	SRR1946541
SRR7648868	SRR1945606	SRR1945918	SRR1946230	SRR1946542
SRR7648873	SRR1945607	SRR1945919	SRR1946231	SRR1946543

SRR7648874	SRR1945608	SRR1945920	SRR1946232	SRR1946544
SRR7648876	SRR1945609	SRR1945921	SRR1946233	SRR1946545
SRR7648877	SRR1945610	SRR1945922	SRR1946234	SRR1946546
SRR7648878	SRR1945611	SRR1945923	SRR1946235	SRR1946547
SRR7648881	SRR1945612	SRR1945924	SRR1946236	SRR1946548
SRR7648882	SRR1945613	SRR1945925	SRR1946237	SRR1946549
SRR7648883	SRR1945614	SRR1945926	SRR1946238	SRR1946550
SRR7648884	SRR1945615	SRR1945927	SRR1946239	SRR1946551
SRR7648885	SRR1945616	SRR1945928	SRR1946240	SRR1946552
SRR7648888	SRR1945617	SRR1945929	SRR1946241	SRR1946553
SRR7648889	SRR1945618	SRR1945930	SRR1946242	SRR1946554
SRR7648890	SRR1945619	SRR1945931	SRR1946243	SRR1946555
SRR7648894	SRR1945620	SRR1945932	SRR1946244	SRR1946556
SRR7648896	SRR1945621	SRR1945933	SRR1946245	SRR1946557
SRR7648897	SRR1945622	SRR1945934	SRR1946246	SRR1946558
SRR7648899	SRR1945623	SRR1945935	SRR1946247	SRR1946559
SRR7648903	SRR1945624	SRR1945936	SRR1946248	SRR1946560
SRR7648906	SRR1945625	SRR1945937	SRR1946249	SRR1946561
SRR7648911	SRR1945626	SRR1945938	SRR1946250	SRR1946562
SRR7648917	SRR1945627	SRR1945939	SRR1946251	SRR1946563
SRR7648918	SRR1945628	SRR1945940	SRR1946252	SRR1946564
SRR7648919	SRR1945629	SRR1945941	SRR1946253	SRR1946565
SRR7648921	SRR1945630	SRR1945942	SRR1946254	SRR1946566
SRR7648922	SRR1945631	SRR1945943	SRR1946255	SRR1946567
SRR7648925	SRR1945632	SRR1945944	SRR1946256	SRR1946568
SRR7648926	SRR1945633	SRR1945945	SRR1946257	SRR1946569
SRR7648928	SRR1945634	SRR1945946	SRR1946258	
SRR7648965	SRR1945635	SRR1945947	SRR1946259	
SRR7648975	SRR1945636	SRR1945948	SRR1946260	
SRR7648976	SRR1945637	SRR1945949	SRR1946261	
SRR7648980	SRR1945638	SRR1945950	SRR1946262	
SRR7648981	SRR1945639	SRR1945951	SRR1946263	
SRR7648982	SRR1945640	SRR1945952	SRR1946264	
SRR7649002	SRR1945641	SRR1945953	SRR1946265	
SRR7649010	SRR1945642	SRR1945954	SRR1946266	
SRR7649012	SRR1945643	SRR1945955	SRR1946267	
SRR7649016	SRR1945644	SRR1945956	SRR1946268	
SRR7649028	SRR1945645	SRR1945957	SRR1946269	
SRR7649033	SRR1945646	SRR1945958	SRR1946270	
SRR7649038	SRR1945647	SRR1945959	SRR1946271	
SRR7649040	SRR1945648	SRR1945960	SRR1946272	
SRR7649041	SRR1945649	SRR1945961	SRR1946273	
SRR7649042	SRR1945650	SRR1945962	SRR1946274	
SRR7649044	SRR1945651	SRR1945963	SRR1946275	

SRR7649045	SRR1945652	SRR1945964	SRR1946276
SRR7649055	SRR1945653	SRR1945965	SRR1946277
SRR7649063	SRR1945654	SRR1945966	SRR1946278
SRR7649064	SRR1945655	SRR1945967	SRR1946279
SRR7649065	SRR1945656	SRR1945968	SRR1946280
SRR7649066	SRR1945657	SRR1945969	SRR1946281
SRR7649067	SRR1945658	SRR1945970	SRR1946282
SRR7649068	SRR1945659	SRR1945971	SRR1946283
SRR7649069	SRR1945660	SRR1945972	SRR1946284
SRR7649070	SRR1945661	SRR1945973	SRR1946285
SRR7649071	SRR1945662	SRR1945974	SRR1946286
SRR7649072	SRR1945663	SRR1945975	SRR1946287
SRR7649074	SRR1945664	SRR1945976	SRR1946288
SRR7649075	SRR1945665	SRR1945977	SRR1946289
SRR7649076	SRR1945666	SRR1945978	SRR1946290
SRR7649081	SRR1945667	SRR1945979	SRR1946291
SRR7649082	SRR1945668	SRR1945980	SRR1946292
SRR7649083	SRR1945669	SRR1945981	SRR1946293
SRR7649086	SRR1945670	SRR1945982	SRR1946294
SRR7649087	SRR1945671	SRR1945983	SRR1946295
SRR7649088	SRR1945672	SRR1945984	SRR1946296
SRR7649090	SRR1945673	SRR1945985	SRR1946297
SRR7649092	SRR1945674	SRR1945986	SRR1946298
SRR7649093	SRR1945675	SRR1945987	SRR1946299
SRR7649094	SRR1945676	SRR1945988	SRR1946300
SRR7649095	SRR1945677	SRR1945989	SRR1946301
SRR7649096	SRR1945678	SRR1945990	SRR1946302
SRR7649097	SRR1945679	SRR1945991	SRR1946303
SRR7649098	SRR1945680	SRR1945992	SRR1946304
SRR7649099	SRR1945681	SRR1945993	SRR1946305
SRR7649100	SRR1945682	SRR1945994	SRR1946306
SRR7649101	SRR1945683	SRR1945995	SRR1946307
SRR7649102	SRR1945684	SRR1945996	SRR1946308
SRR7649103	SRR1945685	SRR1945997	SRR1946309
SRR7649104	SRR1945686	SRR1945998	SRR1946310
SRR7649105	SRR1945687	SRR1945999	SRR1946311
SRR7649106	SRR1945688	SRR1946000	SRR1946312
SRR7649107	SRR1945689	SRR1946001	SRR1946313
SRR7649108	SRR1945690	SRR1946002	SRR1946314
SRR7649111	SRR1945691	SRR1946003	SRR1946315
SRR7649112	SRR1945692	SRR1946004	SRR1946316
SRR7649113	SRR1945693	SRR1946005	SRR1946317
SRR7649114	SRR1945694	SRR1946006	SRR1946318
SRR7649115	SRR1945695	SRR1946007	SRR1946319

SRR7649116	SRR1945696	SRR1946008	SRR1946320
SRR7649117	SRR1945697	SRR1946009	SRR1946321
SRR7649118	SRR1945698	SRR1946010	SRR1946322
SRR7649119	SRR1945699	SRR1946011	SRR1946323
SRR7649120	SRR1945700	SRR1946012	SRR1946324
SRR7649121	SRR1945701	SRR1946013	SRR1946325
SRR7649122	SRR1945702	SRR1946014	SRR1946326
SRR7649123	SRR1945703	SRR1946015	SRR1946327
SRR7649124	SRR1945704	SRR1946016	SRR1946328
SRR7649125	SRR1945705	SRR1946017	SRR1946329
SRR7649126	SRR1945706	SRR1946018	SRR1946330
SRR7649127	SRR1945707	SRR1946019	SRR1946331
SRR7649128	SRR1945708	SRR1946020	SRR1946332
SRR7649129	SRR1945709	SRR1946021	SRR1946333
SRR7649130	SRR1945710	SRR1946022	SRR1946334
SRR7649132	SRR1945711	SRR1946023	SRR1946335
SRR7649133	SRR1945712	SRR1946024	SRR1946336
SRR7649134	SRR1945713	SRR1946025	SRR1946337
SRR7649135	SRR1945714	SRR1946026	SRR1946338
SRR7649136	SRR1945715	SRR1946027	SRR1946339
SRR7649137	SRR1945716	SRR1946028	SRR1946340
SRR7649138	SRR1945717	SRR1946029	SRR1946341
SRR7649139	SRR1945718	SRR1946030	SRR1946342
SRR7649140	SRR1945719	SRR1946031	SRR1946343
SRR7649141	SRR1945720	SRR1946032	SRR1946344
SRR7649142	SRR1945721	SRR1946033	SRR1946345
SRR7649143	SRR1945722	SRR1946034	SRR1946346
SRR7649144	SRR1945723	SRR1946035	SRR1946347
SRR7649145	SRR1945724	SRR1946036	SRR1946348
SRR7649146	SRR1945725	SRR1946037	SRR1946349
SRR7649147	SRR1945726	SRR1946038	SRR1946350
SRR7649148	SRR1945727	SRR1946039	SRR1946351
SRR7649149	SRR1945728	SRR1946040	SRR1946352
SRR7649150	SRR1945729	SRR1946041	SRR1946353
SRR7649151	SRR1945730	SRR1946042	SRR1946354
SRR7649152	SRR1945731	SRR1946043	SRR1946355
SRR7649153	SRR1945732	SRR1946044	SRR1946356
SRR7649154	SRR1945733	SRR1946045	SRR1946357
SRR7649156	SRR1945734	SRR1946046	SRR1946358
SRR7649157	SRR1945735	SRR1946047	SRR1946359
SRR7649158	SRR1945736	SRR1946048	SRR1946360
SRR7649159	SRR1945737	SRR1946049	SRR1946361
SRR7649160	SRR1945738	SRR1946050	SRR1946362
SRR7649161	SRR1945739	SRR1946051	SRR1946363

SRR7649188	SRR1945740	SRR1946052	SRR1946364
SRR7741067	SRR1945741	SRR1946053	SRR1946365
SRR7741068	SRR1945742	SRR1946054	SRR1946366
SRR7784449	SRR1945743	SRR1946055	SRR1946367
SRR7784450	SRR1945744	SRR1946056	SRR1946368
SRR7784451	SRR1945745	SRR1946057	SRR1946369
SRR7818935	SRR1945746	SRR1946058	SRR1946370

Table S2 Transition and transversion rate in organelle genomes of each species.

**Mutation Type**	**Mitochondrial**			**Plastid**		
	Ginkgo	***Oryza***	***Arabidopsis***	***Ginkgo***	***Oryza***	***Arabidopsis***
AG	79	145	288	27	31	445
CT	125	170	231	29	27	430
AC	30	75	315	38	17	418
AT	4	60	63	8	16	311
CG	23	61	94	2	20	199
GT	32	86	303	44	17	482
Transition	204	315	519	56	58	875
Transversion	89	282	775	92	70	1,410
Total	293	597	1,294	148	128	2,285
